# The effect of maternal anaemia on low birth weight among newborns in Northwest Ethiopia

**DOI:** 10.1038/s41598-022-19726-z

**Published:** 2022-09-10

**Authors:** Melaku Tadege Engidaw, Tahir Eyayu, Tegenaw Tiruneh

**Affiliations:** 1 Department of Public Health, College of Health Sciences, Debre Tabor University, P.o.Box: 031, Debre Tabor, Ethiopia; 2 Department of Medical Laboratory Sciences, College of Health Sciences, Debre Tabor University, P.o.Box: 272, Debre Tabor, Ethiopia

**Keywords:** Diseases, Health care, Medical research, Risk factors

## Abstract

Low birth weight is an indicator of maternal-related multifactorial problems such as malnutrition, illness, and work overload. As a result, low birth weight is associated with maternal anaemia, and both of them were significant public health issues in developing nations. Low birth weight and anaemia are caused by insufficient nutrient intake, which is especially severe during pregnancy. So, this study aimed to assess the effect of maternal anaemia during the late trimester on low birth weight among newborns in Northwest Ethiopia. A systematic random sampling technique was used to select 211 participants for the primary data collection. Face-to-face interviews were used to collect data, while blood samples were collected using standard operating procedures. For further analysis, the data file was imported into Stata version 16 (MP) software. The binary logistic regression model was used to investigate significant factors related to low birth weight. Finally, the statistical significance of the variables was determined using a *p* value of ≤ 0.05. The prevalence of anaemia among pregnant women in the late trimester and newborns was 34 (16.11%, 95% CI: 11.42, 21.78) and 64 (30.33%, 95% CI: 24.20, 37.01), respectively. The mean ± standard deviation of the newborn babies' weight was 3.19 ± 0.49 kg. The proportion of low birth weight among newborns was 26 (12.32%, 95% CI: 8.20, 17.53%). The independent effect of anaemia on low birth weight was 4.19 times while all other factors were constant (COR = 4.19, 95% CI: 1.70, 10.30). Maternal educational status [unable to read and write (AOR = 10.94, 95% CI: 1.74, 68.58) and attained secondary education (AOR = 8.06, 95% CI: 1.53, 42.36)], and maternal anaemia (AOR = 3.51, 95% CI: 1.29, 9.55) were associated with low birth weight after adjusting with all other variables. In this study, the proportion of low birth weight was high. Here, maternal anaemia alone had a significant independent role in the development of low birth weight. Maternal education status and anaemic conditions were associated with low birth weight among newborns. Early detection and treatment of maternal anaemia during pregnancy is crucial with the usual nutritional-related care.

## Introduction

Anaemia is hematological abnormality and has a significant public health problem globally. World Health Organization (WHO) reports that more than 1.62 billion people are affected globally. Of these, 56 million pregnant women (41.8%) were suffering from varying degrees of anaemia^1^. Despite anaemia being found worldwide, it's more prevalent in developing countries^2^. In Ethiopia, the prevalence of anaemia among pregnant women ranges from 11.6 to 45.4%^3,4^.

Anaemia is one of the complications during pregnancy, and it also has adverse effects on neonatal outcomes like^5^ fetal anaemia, stillbirth, and low and very low birth weight (LBW)^6,7^. A study conducted in Nepal showed that the risk of LBW was 6.8 times higher among anaemic mothers^8^. Another study reveals that anaemia during pregnancy increases the risk of LBW among newborns^9^.

Maternal nutritional deficiency during pregnancy affects the developmental process of the fetus, which subsequently influences the birth weight of the newborn^10^. The fetus is highly dependent on maternal nutritional intake since malnutrition during pregnancy leads to different adverse birth outcomes like LBW. During pregnancy, insufficient storage or inadequate intake of essential nutrients can cause harmful effects on both the mothers and newborn babies^11–13^.

Birth weight is a good indicator to measure multi-layered pubic-related health problems like long-term maternal malnutrition, health status, and poor health care delivery system. The LBW is one of the most basic and common health indicators used to assess the status of infants. It is still the major determinant of mortality, morbidity, and disability in infancy and childhood to a long-term impact on health outcomes in adult life. Also, it affects the health sector economy, has a higher risk of death and illness shortly after birth and non-communicable disease, and is a significant burden on society as a whole^14,15^.

According to the WHO, we can say LBW if the weight of the newborn is < 2500 gm^16^. The prevalence of LBW ranges from 15 to 20% (over 20 million births annually) globally, and a majority (90%) of them are found in low-and-middle-income countries^17,18^. According to the Ethiopian demographic health survey (EDHS) report, the magnitude of LBW increased from 11 to 13%^19,20^. Also, this prevalence is high (18%) in Southern Ethiopia^21^. Based on the 2011 EDHS finding, the magnitude of the LBW report varies from region to region, 30% in Afar, 28% in Amhara, 26% in Somali, and 27% in the Gambela region^19^.

As corresponded to normal, LBW infants were 20 times more likely to develop complications and die^22^. It is the potential risk of cognitive deficits^23^, motor delays^24^, cerebral palsy^25^, other behaviour^26^, and psychological-related problem^27^.

Birth weight is one of the predictive factors of newborn death in the first few months of life^28^. In Ethiopia, in 2014, there were 27,243 deaths, of which LBW accounted for 4.53% of the total deaths^29^. Globally, LBW is still a significant determinant factor for infant mortality, and morbidity, which causes short- and long-term consequences in later life.

LBW is an adverse health outcome that continues to later life and seriously impairs the normal functioning of an individual. LBW comes with numerous social and economic problems for a country^30^. Therefore, this study aimed to assess the effect of maternal anaemia on the birth weight of newborn babies.

## Materials and methods

### Study design, period, and setting

The primary data were collected cross-sectionally from February 1 to April 30, 2019. The initial study was conducted at the Gynecology and Obstetrics Department, University of Gondar Comprehensive Specialized Hospital in Gondar Town, Northwest Ethiopia. The Town is located in the Amhara region, 180 km far from Bahir Dar, the capital city of the Amhara regional state, and 727 km from Addis Ababa, the capital city of Ethiopia. Gondar Town is found 2133 m above sea level. The hospital delivers several primary services in paediatrics, surgery, gynaecology and obstetrics, and Internal medicines departments with teaching–learning activities.

### Study population

All full-term newborn babies (39–42 weeks of gestational age) were the study population for this secondary data analysis.

### Sample size determination

For the primary objective (i.e. prevalence and associated factors of anemia among full-term newborn babies), the sample was determined using a single population proportion formula. But, this is a secondary data analysis beyond the primary objective. So, this secondary data analysis includes 211 full-term newborn babies data^31^.

### Eligibility criteria

For this study, 211 study participants' data were included (full-term newborn babies paired with their mothers). During primary data collection, mothers who had twin newborns, complications during pregnancy, and known chronic illnesses (diabetes, preeclampsia/hypertension, HIV/AIDS, chronic kidney, liver disease, malaria, and any malignancy) were excluded from this study. If the clinician had already confirmed the diagnosis, then the presence of co-morbidities was taken into account (subclinical conditions were not investigated). Moreover, individuals with any hemoglobinopathy conditions were excluded from the study.

### Sampling procedure

This secondary data was collected by applying a systematic random sampling technique was employed to select study participants. At this time, every 4^th^ pregnant woman during the delivery time from February 1 to April 30, 2019. The first study participant was selected randomly by lottery methods every day, and excluded study participants were substituted by the succeeding participants (subsequent consecutive study participants).

### Data collection methods and tools

At the time of primary data collection, the pre-tested Amharic version questionnaires were employed. The questionnaire consists of socioeconomic, dietary intake (iron-rich foods), antenatal care (ANC) follow-up, supplementation, complication during birth, and previous pregnancy-related characteristics. The weights were measured using the Seca scale, and results were recorded to the nearest 0.1 kg. About 3 ml cord blood sample was drawn from the umbilical vein of the cord by midwives professional, and from each selected mother, 3 ml of blood was drawn from each mother. Then, a complete blood count was done by laboratory professionals. The dietary data were collected using a meal frequency questionnaire with five options (once a week, twice a week, more than twice a week, once per month, and less than one time per month). Finally, we reduced it into three categories due to small frequency (twice and above a week, once a week, and no/never/not at all).

### Data quality

Initially, a pre-test was done on 5% of the total sample size outside the study area (at Debre Tabor Referral Hospital) after training the data collectors. In addition, the questionnaire was collected using the Amharic language (local). At all times during the primary data collection, cross-checking of collected questionnaires was done daily. The collected data were checked for completeness and consistency by investigators and supervisors.

To ensure the quality of the laboratory test, a standard operating procedure was followed during specimen collection, and daily cleaning and background runs were done to ensure the laboratory results. Also, to avoid hemolysis and mix-up of sample, appropriate sample transportation using a test tube and labelling was done on the sample with the same identification number. All materials and reagents were checked for the expired date before sample processing.

### Operational definitions

- **Anaemia among newborns**: If the newborn Hb level was < 14 mg/dl from newborn cord blood.
- **Anaemia among pregnant women**: If a Hb level of a pregnant woman was < 11 mg/dl after adjusting for altitude. The magnitude is further categorized as mild, moderate and severe anaemia if the haemoglobin count (g/dl) is between 10.0 and 10.9, 7.0 and 9.9 and < less than 7.0, respectively.
- **Low birth weight:** If the birth weight of a newborn was < 2500 gr.

### Data processing and analysis

This secondary data was imported to the Stata. Then, by using the Stata 16.0/MP version for windows, data cleaning, recategorizing, and tabulation were done. Then, both descriptive and analytical analysis of variables was employed. Binary and multivariable binary logistic regression analysis was hired to assess the independent and multiple effects of each variable on the LBW. During bivariable binary logistic regression, candidate variables for multivariable analysis were chosen if the *p* value was ≤ 0.25. Finally, a *p* value ≤ 0.05 was used to declare statistical significance during multivariable analysis. For each odd ratio (ORs; adjusted and crude), 95% CI was computed. The model fitness was tested using Hosmer and Lemeshow tests (*p* value = 0.9711) during the final model.

### Ethical approval and consent from participants

For this secondary data analysis, the ethical clearance letter was obtained from the Ethical Review Committee of Debre Tabor University. Also, written informed consent was obtained from each selected mother after explaining the study. The data was collected without personal identifiers, and for anaemic and low-birth newborn babies, immediate consultation took place in addition to information dissemination by data collectors.

## Results

### Sociodemographic results of study participants

In this study, 211 participants' data were analyzed. The mean ± SD of the pregnant women’s age was 27.36 ± 5.19 years, which ranges from 18 to 44 years. All of the participants were from Amhara ethnic. Of all, 178 (84.36%) pregnant women were from urban areas. Only one-third of the pregnant women attended more than secondary school and above (Table 1).

**Table 1:** Sociodemographic characteristics of the study participant of the pregnant mother and the newborns Northwest Ethiopia, 2019 (n = 211).

Characters	Category	Frequency	Percentage (%)
Age (years)	16–19	26	12.32
	20–24	56	26.54
	25–29	90	42.65
	≥ 30	39	18.48
Religion	Orthodox	188	89.10
	Muslim	20	9.48
	Protestant	3	1.42
Residence	Urban	178	84.36
	Rural	33	15.64
Educational status	Unable to read and write	36	17.06
	Able to read and write	44	20.85
	Primary school (1–8)	61	28.91
	Secondary school and above	70	33.18
Occupational status	Employed	70	33.18
	Housewife	141	66.82
Sex of the newborns	Male	106	50.24
	Female	105	49.76

### Maternal reproductive health and newborn-related characteristics

Almost all pregnant women [198 (93.84%)] had ANC follow-up for their respective pregnancies. Also, all of the mothers had an intake history of Iron with folic acid supplementation for this pregnancy. More than half of the pregnant women [118 (55.92%)] were multigravida, while others were primigravida [93 (44.08%)]. From all, 171 (81.04%) the index children were delivered vaginally, but the rest of the pregnant women were delivered via cesarean section. Of all pregnant women, only [18 (8.53%)] have had a history of vaginal bleeding during their pregnancy.

### Dietary intake-related characteristics of the pregnant women

Among study participants, the consumption of iron reach foods at least once per week was 50%. The consumption of vegetables, fruits, and red meat once per week was 93 (44.08%), 110 (52.13%), and 133 (63.03%), correspondingly (Table 2).

**Table 2:** Dietary habit-related characteristics of pregnant women in Northwest Ethiopia, 2019 (n = 211).

Characters	Category	Frequency	Percentage (%)
Consumption vegetable	Twice a week and above	76	36.02
	Once a week	93	44.08
	Never	42	19.91
Consumption fruit	Twice and above a week	55	26.07
	Once a week	110	52.13
	No/never	46	21.80
Consumption of red meat	Once a week	133	63.03
	Not at all	78	36.97

### The magnitude of anaemia and low birth weight

The mean ± SD of the pregnant women's Hb after adjusting for altitude and hematocrit was 13.23 ± 1.81 g/dl and 39.66 ± 5.30, correspondingly. The prevalence of anaemia among the delivered women using the adjusted Hb for altitude was 34 (16.11%, 95% CI: 11.42, 21.78). Among anaemic pregnant women, the magnitude of moderate and mild anaemia was 11 (5.21%) and 23 (10.90%), respectively.

The mean ± SD of the newborn babies' Hb and hematocrits was 15.70 ± 2.11 g/dl and 47.81 ± 6.06, correspondingly. The prevalence of anaemia among the newborn was 64 (30.33%, 95% C:I 24.20, 37.01) using the cord blood adjusted Hb for altitude. The mean ± SD of the newborn babies’ weight was 3.19 ± 0.49 kg, which ranges from 1.60 to 4.2kgs. The proportion of LBW among newborns was 26 (12.32%, 95% CI: 8.20, 17.53%). The prevalence of LBW among anaemia and non-anaemic women was 10 (29.41%, 95% CI: 15.09–47.7%) and 16 (9.04%, 95% CI: 5.25 - 14.26%), respectively.

### Factors associated with low birth weight among newborn

While all other factors were assumed constant, pregnant women who had anaemia during late pregnancy were 4.19 (COR = 4.19, 95% CI: 1.70, 10.30) times more likely to have a newborn with LBW as compared to those who had no anaemia. This finding shows that the prevention of anaemia is vital to decreasing the occurrence of LBW.

Also, the adjusted effects were assessed using bi-variable binary logistic regression. At this time, maternal anaemia status, maternal educational status, maternal occupation, consumption habit of vegetables, and consumption habit of red meat were the significantly associated variables for LBW. Finally, these significant variables were fitted into multivariable binary logistic regression. During multivariable analysis, maternal anaemia status and maternal educational status were the associated factors for the occurrence of LBW among newborns as shown in the table below (Table 3).

**Table 3:** Binary and multivariable logistic regression analysis of factors associated with low birth weight among newborns in Northwest Ethiopia, 2019 (n = 211).

Variables	LBW		Odds ratios	
	Yes	No	COR (95% CI)	AOR (95% CI)
**Maternal educational status**				
Unable to read and write	8	28	**9.71 (1.94, 48.63)**	**10.94 (1.74, 68.58)**
Primary school 1–8)	5	39	4.35 (0.81, 23.53)	5.35 (0.84, 34.01)
Secondary school	11	50	**7.48 (1.58, 35.25)**	**8.06 (1.53, 42.36)**
Above secondary school	2	68	1	**1**
**Maternal occupation**				
Employed	6	64	1	1
Housewives	20	121	1.76 (0.67, 4.61)	0.56 (0.17, 1.79)
**Consumption of vegetables**				
Twice a week and above	4	35	0.28 (0.07, 1.01)	0.46 (0.11, 1.98)
Once a week	15	78	0.96 (0.36, 2.56)	1.47 (0.46, 4.64)
Never	7	72	1	1
**Consumption of red meats**				
Yes	7	71	1.69 (0.67, 4.22)	1.88 (0.70, 5.03)
No	19	114	1	1
**Maternal anaemia status**				
No	16	161	**4.19 (1.71, 10.30**)	3.51 (1.29, 9.55) *
Yes	10	24	1	1

LBW, low birth weight; *shows statistically significant *p* < 0.05; 1 = reference group; AOR, adjusted odd ratio; COR, crude odd ratio.

Significant values are in bold.

In this study, newborn babies from unable to read and write pregnant women were 10.94 times more likely (AOR = 10.94, 95% CI: 1.74, 68.58) to have LBW babies as compared to those who attend above high school. Also, these mothers who attended secondary education were 8.06 times (AOR = 8.06, 95% CI: 1.53, 42.36) more likely to give LBW newborn.

Likewise, the independent effect above, newborn children from anaemic pregnant women were 3.51 times (AOR = 3.51, 95% CI: 1.29, 9.55) more likely to have a low-birth-weight baby as compared to non-anaemic pregnant women.

## Discussion

LBW is a leading public health problem that is mainly associated with an increased risk of newborn morbidity and mortality. Among different causes of LBW, anaemia during pregnancy is the one, which is occurred in each trimester of pregnancy worldwide^32–37^.

The prevalence of anaemia among the delivered women was 16.11% (95% CI: 11.42, 21.78). The result in this study is low while we compared with a study done in Jimma, Ethiopia (27.4%)^38^, Northwest Ethiopia (25.2%)^39^, India (78.45%)^40^, Egypt (72%)^41^, and Turkey (27%)^42^. This variation might be due to eligibility criteria like pregnancy complicated with diabetics, preeclampsia, hypertension, HIV/AIDS, and malaria were considered in these studies but excluded in our study. In addition, participants' habits of consumption of vegetables (80.1%), fruit (78%)^43^, and red meat (63%) were high. Besides, the regular antenatal follow-up with Iron and Folic Acid supplementation during pregnancy is also an asset^44,45^. All these factors were essential to prevent the occurrence of anaemia during pregnancy.

In this study, the prevalence of anaemia among newborns was 30.33% (95% CI: 24.20, 37.01) which was comparable to a study conducted in Brazil (32.6%)^46^. The possible reasons might be a similarity in socioeconomic status, dietary habits, lack of supplementation during pregnancy, and poor preconception and conception care.

The proportion of the LBW in the current study was 12.32%, (95% CI: 8.20, 17.53%). The finding of this study was comparable to the study done in Northern Ethiopia (10%)^37^, Pakistan (10.6%)^47^, Iran (9.5%)^48^, Northwest Ethiopia (14.9%)^49^, and Dessie, Ethiopia (15.6%)^50^. However, in this study, the prevalence of LBW was higher than in the study conducted in Northwestern Iran (6.8%)^51^. This might be due to the presence of maternal morbidity like anaemia secondary to inadequate intake of micro and macronutrients during pregnancy. In addition, maternal weight gain during pre-pregnancy is also the most crucial factor. Mothers' nutritional status is the most significant determinant of newborn children's birth weight. In this study, anaemic mothers were more likely to deliver LBW than those who had no anaemia, which was consistent with a study finding in India^52^.

The prevalence of LBW was lower as compared to a study conducted in Dilla Town, Southern Ethiopia (34.1%), Eastern Ethiopia (21%)^53^, India (22%)^52^, and Nigeria (20.3%)^54^. These might be due to sample size, dietary behaviours, and access to maternal health services on time, early ANC follow-up, preconception level of awareness, conception care, and household's asset or wealth status.

Here, being a newborn from a mother with anaemia alone will increase the risk of LBW by four times (COR = 4.19 (95% C:I 1.71, 10.30) as compared to their counterparts. Even this risk will increase more than 3-folds (AOR = 3.51 (95% CI: 1.29, 9.55) with all other contributing factors. The result indicates that prevention and controlling of anaemia alone will play a significant role to prevent the occurrence of LBW among newborns. The finding of this study is in line with the study conducted in Nigeria^54^, Indonesia^55^, Northern Ethiopia^56^, Southern Ethiopia^57^, and Dessie town, Ethiopia^58^. Another study in India also showed that maternal anaemia increases the incidence of LBW babies by 6.5%^59^. These might be due to the occurrence of anaemia secondary to hemodilution physiologically. Moreover, anaemia during pregnancy might be happening due to inadequate intake of nutrients, unable to take the recommended dose of Iron with folic acid supplementation during pregnancy, poor preconception and conception care, morbidity during pregnancy like helminthiasis, and poor diet quality. All these factors will directly or indirectly cause LBW.

In our study, newborn babies born from mothers who were unable to read and write were 10.94 times more likely (AOR = 10.94, 95% C:I 1.74, 68.58) to have LBW babies compared to those who attend above high school. Additionally, these mothers who attended secondary education were 8.06 times (AOR = 8.06, 95% CI: 1.53, 42.36) more likely to give LBW newborn. This finding is consistent with a study conducted in Ethiopia^60^. But maternal education was not statistically significant in the other studies conducted in two different parts of Ethiopia^49,53^. The possible reasons might be the level of awareness related to preconception care, residence, and the socioeconomic status of being unable to read and write might not be different.

Finally, maternal anaemia had significant effects of on the newborn birth weight. This study’s findings are similar to those study reported in China^61^, India^40^, Brazil^62^, Nepal^63^ Colombia^64^, Eastern Ethiopia^65^, and Southern Punjab, Asia^66^. This could be because of intrauterine growth restriction. In a low oxygen environment, the placenta proliferates and grows. When the maternal hemoglobin level falls, oxygen circulation in the fetal body is restricted. The fetal placenta is thus exposed to an oxidative stress environment (chronic hypoxia). As, a result Intrauterine fetal hypoxia impairs the transfer of oxygen/nutrient supply, resulting in fetal growth restriction and low birth weight^67,68^ due to placental angiogenesis.

## Limitation of the study

Since this is secondary data analysis, all determinant variables were not fully considered in this study. This study might not link specific macro and micro-nutrient deficiencies as a cause of anaemia, a particular type of anaemia, and difficult to establish a possible causality with LBW. Furthermore, this study did not include the assessment of subclinical conditions at the time of the primary data collection, and some essential laboratory tests (like stool exams) were not done.

## Conclusion

The prevalence of anaemia among pregnant women was a moderate public health problem. The magnitude of LBW among newborns was relatively high. Anaemia during pregnancy is the most significant predictor of LBW alone. Also, maternal educational status is one of the factors affecting LBW.

According to this study, during routine care, treatment of anaemia is highly recommended with regular Iron Folic Acid supplementation. Also, regular nutrition education and counselling are essential based on the maternal educational status. The involvement of stakeholders in the treatment of anaemia is warranted to decrease the rate of LBW and its late consequence.

## Data Availability

All the important data is found within the manuscript.

## Abbreviations

ANC: Antenatal care
AOR: Adjusted odds ratio
COR: Crude odds ratio
Hb: Haemoglobin
LBW: Low birth weight
SD: Standard deviation
WHO: World Health Organization
